# Solitary Metastasis From Cutaneous Melanoma to the Liver: Resection by Extended Left Hepatectomy (Trisegmentectomy) With Clearance of Tumor From the Portal Vein

**DOI:** 10.1155/1994/29169

**Published:** 1994

**Authors:** Jean-Nicolas Vauthey, Matthias W. Winter, Leslie H. Blumgart

**Affiliations:** 1 Department of Surgery Memorial Sloan-Kettering Cancer Center New York N.Y. USA; 2 Department of Surgery P.O. Box 100286 University of Florida College of Medicine Gainesville Florida 32610-0286 USA

## Abstract

A 61-year-old woman presented with low grade fever and an epigastric mass eight years following resection of a stage Clark IV infraclavicular cutaneous melanoma followed by axillary node dissection. Investigations revealed a tumor in segment II, III, IV and V of the liver and a thrombus involving the main portal vein. Liver resection with extended left hepatectomy (left trisegmentectomy) and portal vein thrombectomy is reported.


*HPB Surgery*, 1994, Vol. 8, pp. 53–56
Reprints available directly from the publisher
Photocopying permitted by license only

© 1994 Harwood Academic Publishers GmbH
Printed in Malaysia

### Solitary Metastasis from Cutaneous Melanoma to the Liver: Resection by Extended Left Hepatectomy (Trisegmentectomy) with Clearance of Tumor from the Portal Vein

JEAN-NICOLAS VAUTHEY*, MATTHIAS W. WINTER and LESLIE H. BLUMGART

Department of Surgery, Memorial Sloan-Kettering Cancer Center, New York, N.Y.

A 61-year-old woman presented with low grade fever and an epigastric mass eight years following resection of a stage Clark IV infraclavicular cutaneous melanoma followed by axillary node dissection. Investigations revealed a tumor in segment II, III, IV and V of the liver and a thrombus involving the main portal vein. Liver resection with extended left hepatectomy (left trisegmentectomy) and portal vein thrombectomy is reported.

KEY WORDS: Melanoma tumor thrombus thrombectomy liver resection extended resection trisegmentectomy

### INTRODUCTION

Metastatic cutaneous melanoma to the liver usually presents as multiple hepatic recurrences and in association with widespread metastatic disease[1]. In contrast, occular melanoma often recurs in the liver as the only metastatic site after a long disease-free interval[2]. Rarely, hepatic metastases from melanoma are resectable[3].

In this report, a patient with a long disease-free interval following resection of an infraclavicular cutaneous melanoma and axillary node dissection presented with a single liver metastasis and carcinomatous portal vein thrombosis. Resection with extended left hepatectomy (left trisegmentectomy) and portal vein reconstruction is reported.

* *Present Address*: Jean-Nicolas Vauthey, MD, Assistant Professor Department of Surgery, P.O. Box 100286, University of Florida College of Medicine, Gainesville, Florida 32610-0286, USA.

### CASE REPORT

A 61 year old woman presented with low grade fever, myalgia and a 6 cm epigastric mass. Six years prior to this, she had had resection of a Clark's IV infraclavicular cutaneous melanoma and an axillary node dissection with 3 out of 40 positive nodes. At presentation, laboratory tests were significant for raised lactic dehydrogenase (LDH) (766 U/L, normal value 60 to 200 U/L). Alphafetoprotein (AFP) was 3.1 ng/ml (normal value 0.0 to 8.5 ng/ml). Hepatitis *B* and *C* serology were negative. Dynamic computed tomography (CT) showed a mass in the left liver extending to involve segment V of the right liver. CT of the head and chest showed no evidence of metastatic disease. CT portography showed hypoperfusion and a mass in the left lobe of the liver with extension into segment V of the right lobe and a portal vein thrombus (Figure 1). Selective mesenteric arteriography shown in Figure 2 reveals replacement of the right hepatic artery with origin from the superior mesenteric artery.

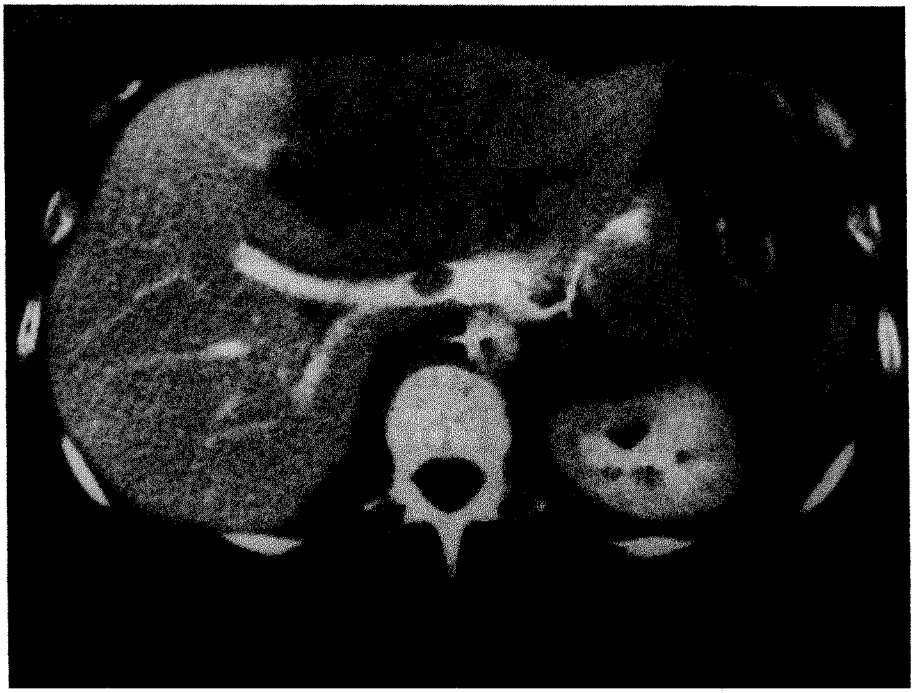

**Figure 1** CT portography illustrating tumor involving segments II, III, IV and V and left portal vein thrombus extending into main portal vein (arrow).

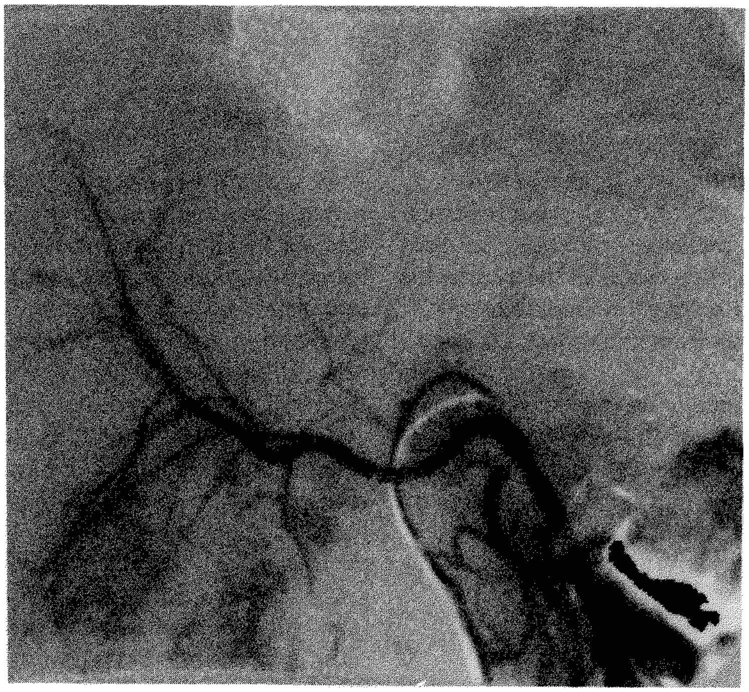

**Figure 2** Selective superior mesenteric arteriography showing replaced right hepatic artery arising from superior mesenteric artery with anterior branch supplying part of the tumor. The remaining liver is supplied by anatomically normally arising left hepatic artery (not shown).

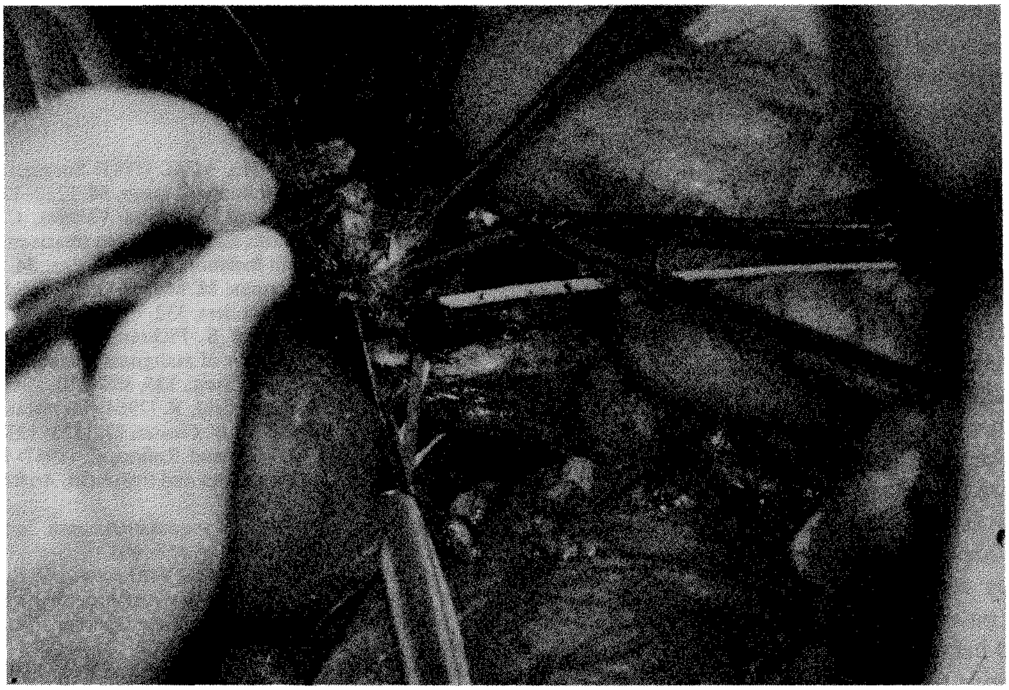

**Figure 3** Extraction of thrombus (arrow).

An extended left hepatectomy (left trisegmentectomy) was performed. The procedure consisted of removal of segments II, III, IV, V and VIII. The origin of the left portal vein was divided after vascular control was obtained by clamping the main portal vein and right portal vein. The thrombus extending into the main portal vein was extracted by a venotomy at the bifurcation of the main and left portal vein and the portal vein was reconstructed (Figure 3). The left and middle hepatic veins were divided extrahepatically. The vascularly isolated left liver along with segments V and VIII were then resected in a plane anterior to the right portal scissure securing the anterior sectoral branch of the right portal pedicle within the liver substance. Blood loss amounted to 450 cc during mobilization of the liver and 550 cc during parenchymal dissection. The patient received a total of 2 units of autologous blood perioperatively. Pathology showed bilobar metastatic melanoma with left portal vein tumor thrombus. Hepatic and portal vein resection margins were negative. Lymph nodes from the hilus of the liver showed no evidence of malignancy.

The patient was discharged on postoperative day 14 with a biliary fistula draining less than 200 ccs per day. HIDA-scan showed a minor biliary fistula with normal uptake of the isotope in the remaining liver and unimpaired excretion into the intestine. Bile drainage subsided spontaneously on postoperative day 34.

### DISCUSSION

The liver is the only site of extraregional metastases in 1 to 4% of patients with cutaneous melanoma[4,5,6], but advanced melanoma is known to widely metastasize to multiple sites[7]. Autopsy studies have shown diffuse hepatic liver involvement in 52 to 68% of advanced metastatic cutaneous melanoma[4,5,8] while liver failure is a cause of death in 7%[4].

Isolated liver involvement by metastatic cutaneous melanoma is rare but occular melanoma has a unique pattern of isolated metastasis to the liver following a long disease-free interval[2]. In this case, a portal vein thrombosis initially suggested primary hepatocellular carcinoma. While hepatic resection can provide long-term survival in hepatocellular carcinomas, survival after resection of isolated hepatic metastases from cutaneous melanoma is unknown.

Comparison between chemotherapy and resection of isolated distant metastases from cutaneous melanoma shows a better palliation and prolonged survival after resection[1,9]. After complete resection of a single extraregional metastasis from cutaneous melanoma, a long-term survival of 20 to 25% is expected[10]. Caputy *et al.*[11] found that a disease-free interval of longer than 2 years between treatment of the primary melanoma and the development of gastrointestinal metastases was associated with improved survival.

Presence or absence of nodal metastases at the time of primary melanoma diagnosis had no significant impact on the duration of patient survival once gastrointestinal metastases developed. Branum *et al.*[12] reported on gastrointestinal metastases and found that if all known disease was resected, median survival was 17 months. Incomplete resection had a median survival of 7 months comparable to no resection and bypass procedures.

In this patient, the metastasis was isolated and completely resectable by extended left hepatectomy (trisegmentectomy) and portal vein thrombectomy. Extended left hepatectomy is a complex procedure with the attending risks of heavy blood loss and bile duct injuries[13]. The resection line is anterior to the right portal scissure in a plane parallel to the right hepatic vein[14,15]. Blood loss during the procedure results mainly from hepatic venous bleeding. It can be greatly minimized by complete extrahepatic vascular isolation. In this patient, the left and middle hepatic vein were isolated before parenchymal transection and additional margins were obtained by dividing the anterior sectoral branch of the right portal pedicle within the liver substance as recently reported[15,16].

Portal vein thrombosis occurs in association with cirrhosis, neoplasm, infection, inflammation (pancreatitis), myeloproliferative disorders or idiopathically[17]. Carcinomatous thrombosis commonly occurs in association with advanced pancreatic carcinoma and hepatocellular carcinoma secondary to direct portal vein invasion by tumor or extrinsic compression of the portal vein[18,19,20]. Carcinomatous thrombosis from metastases is uncommon[17] and in this case the association with melanoma is unusual. Patients with malignant portal vein thrombosis are less likely to survive long enough to develop the sequelae of portal hypertension and variceal hemorrhage is rare[21]. Exceptionnally, portal vein thrombi are resectable. Techniques of portal vein tumor thrombus extraction include balloon catheter extraction, portal vein resection with direct reconstruction or grafting and removal of tumor thrombus under direct vision as in this case. Survival of more than 3 years after portal vein tumor thrombus extraction for hepatocellular carcinoma has been reported[22].

In this patient, a carcinomatous thrombus of the left portal vein from metastatic cutaneous melanoma to the liver extended into the main portal vein without invading the wall of the main portal vein. Thrombectomy along with complete tumor resection and negative margins provided the best palliation and possibly the prospect of long-time survival.

### REFERENCES

1. Overett, T. K. and Shiu, M. H. (1985) Surgical treatment of distant metastatic melanoma. *Cancer*, **56**, 1222–1230.
2. Einhorn, L. H., Burgess, M. A. and Vallejos, C. *et al.* (1974) Prognostic correlations and response to treatment in advanced metastatic malignant melanoma. *Cancer Res.*, **34**, 1995–2004.
3. Foster, J. H., and Berman, M. M., eds. (1977) Solid liver tumors. *Philadelphia*: W. B. Saunders, 332.
4. Patel, J. K., Didolkar, M. S., Pickren, J. W. and Moore, R. H. (1978) Metastatic pattern of malignant melanoma. A study of 216 autopsy cases. *Am J Surg.*, **135**, 807–810.
5. Das Gupta, T. and Brasfield, R. (1964) Metastatic melanoma. A clinicopathological study. *Cancer*, **17**, 1323–1339.
6. Stehlin, J. S., Hills, W. J. and Rufino, C. (1967) Disseminated melanoma. Biologic behavior and treatment. *Arch Surg*, **94**, 495–501.
7. Balch, C. M., ed. (1992) Cutaneous melanoma. 2nd ed. *Philadelphia: JB Lippincott Company*, 458–460.
8. Amer, M. H., Al-Sarraf, M. and Vaitkevicius, V. K. (1979) Clinical presentation, natural history and prognostic factors in advanced malignant melanoma. *Surg Gynecol Obstet*, **149**, 687–692.
9. Lejeune, F. J., Liénard, D., Sales, F. and Badr-El-Din, H. (1992) Surgical management of distant melanoma metastases. *Sem Surg Oncol*, **8**, 381–391.
10. Coit, D. G. and Brennan, M. F. (1993) Current management of malignant melanoma. *Surgery*, **113**, 128–129.
11. Caputy, G. G., Donohue, J. H., Goellner, J. R., and Weaver, A. L. (1991) Metastatic melanoma of the gastrointestinal tract. Results of surgical management. *Arch Surg.*, **126**, 1353–1358.
12. Branum, G. D. and Seigler, H. F. (1991) Role of surgical intervention in the management of intestinal metastases from malignant melanoma. *Am J Surg.*, **162**, 428–431.
13. Starzl, T. E., Iwatsuki, S. and Shaw, B. W., Jr. *et al.* (1982) Left hepatic trisegmentectomy. *Surg Gynecol Obstet.*, **155**, 21–27.
14. Blumgart, L. H. (1988) Liver resection—liver and biliary tumours. In: Blumgart, L. H., ed. Surgery of the liver and biliary tract. *Edinburgh: Churchill Livingstone*, 1251–1280.
15. Blumgart, L. H., Baer, H. U. and Czerniak, A. *et al.* (1993) Extended left hepatectomy: technical aspects of an evolving procedure. *Br J Surg*, **80**, 903–906.
16. Launois, B. and Jamieson, G. G. (1992) The posterior intrahepatic approach for hepatectomy or removal of segments of the liver. *Surg Gynecol Obstet.*, **174**, 155–158.
17. Cohen, J., Edelman, R. R. and Chopra, S. (1992) Portal vein thrombosis: A review. *Am J Med.*, **92**, 173–182.
18. Albacete, R. A., Matthews, M. J. and Saini, N. (1967) Portal vein thromboses in malignant hepatoma. *Ann Intern Med.*, **67**, 337–348.
19. Gold, J. A., Sostman, H. D. and Burrell, M. I. (1979) Cholangiocarcinoma with portal vein obstruction. *Radiology*, **130**, 15–20.
20. Harnar, T., Johansen, K., Haskey, R. and Barker, E. (1982) Left-sided portal hypertension from pancreatic pseudotumor. *Am J Gastroenterol*, **77**, 639–641.
21. Witte, C. L., Brewer, M. L., Witte, M. H. and Pond, G. B. (1985) Protean manifestations of pylethrombosis. A review of thirty-four patients. *Ann Surg.*, **202**, 191–202.
22. Ozawa, K., Takayasu, T. and Kumada, K. *et al.* (1991) Experience with 225 hepatic resections for hepatocellular carcinoma over a 4-year period. *Am J Surg.*, **161**, 677–682.

